# Differential expression of nuclear hormone receptors by dendritic cell subsets in human vaginal mucosa and skin

**DOI:** 10.3389/fimmu.2022.1063343

**Published:** 2023-01-13

**Authors:** HyeMee Joo, Chao Gu, Matthew Wiest, Dorothee Duluc, Emyly Fernandez, Verah Nyarige, Johnny Yi, SangKon Oh

**Affiliations:** ^1^ Department of Immunology, Mayo Clinic, Scottsdale, AZ, United States; ^2^ Immunoconcept, Centre National de la Recherche Scientifique (CNRS) UMR 5164, Bordeaux University, Bordeaux, France; ^3^ Department of Health Sciences Research, Mayo Clinic, Scottsdale, AZ, United States; ^4^ Department of Medical and Surgery Gynecology, Mayo Clinic, Phoenix, AZ, United States

**Keywords:** nuclear hormone receptor, estrogen receptor, progesterone receptor, dendritic cell, antigen-presenting cells, vaginal mucosa, skin, blood

## Abstract

Nuclear hormone receptors (NHRs) expressed by dendritic cells (DCs), the major immune inducers and regulators, could play important roles in host immunity. Assessment of NHRs expressed by DCs in the vaginal mucosa (VM), in comparison with those expressed by DCs in other tissues, will thus help us understand the immunology of human vagina. This study identified 16 NHR transcripts that are differentially expressed among 8 different antigen-presenting cell (APC) subsets isolated from human VM, skin, and blood. The expression profiles of NHRs were largely tissue specific. VM APCs expressed increased levels of *LXRA, RXRA*, *ESRRA, ESRRAP2*, and *PPARG*, whereas skin and blood APCs expressed increased levels of *NURR1, NOR1* and *RARA*. Of interest, female sex hormone receptors, *ESR1* and *PGR*, were found to be mainly expressed by non-APC cell types in the VM; *ESR1* by HLA-DR^+^CD34^+^ and *PGR* by HLA-DR^-^ cells. ERα and PR were expressed by vimentin^+^ cells in the VM, but not in human skin. ERα, but not PR, was also expressed in CD10^+^ cells in the lamina propria of VM. In conclusion, NHR expression by APC subsets is tissue- and cell type-specific. Future studies on the roles of individual NHRs expressed by different cell types, including DC subsets, in the human VM are warranted.

## 1 Introduction

The vaginal mucosa (VM) is the main entry site of a variety of microbial pathogens, including sexually-transmitted pathogens (1, 2). Therefore, VM has long been considered an attractive site for mounting protective immunity. However, the lower female genital tract, including VM, a site constantly exposed to foreign antigens, is also thought to be a unique tolerogenic microenvironment that can tightly regulate unwanted types of immune response (3, 4). Nonetheless, the immunology of the human VM still remains poorly understood.

Dendritic cells (DCs) are the major antigen-presenting cells (APCs) that can induce and control the host immune responses (5). We previously reported that there are 4 major subsets of APCs in the human VM tissues; Langerhans cells (vLC) in the epithelium, CD1c^+^CD14^-^ DCs (vCD14^-^ DC), CD1c^+^CD14^+^ DCs (vCD14^+^ DC), and CD1c^-^CD14^+^ macrophages (vMØ) in the lamina propria (LP) (6, 7). Each of these vaginal APC subsets can display functional specialty in directing T cell responses (6, 7), as do subsets of DCs isolated from human skin (8, 9). We also reported that DCs in the human VM tissues display unique molecular features (10), when compared to DCs in human skin and peripheral blood, which could further impact the magnitude and types of immune response elicited by the individual VM DC subsets.

Nuclear hormone receptors (NHRs) are ligand-activated transcription factors that can recognize a variety of small molecules, including lipids, hormones, vitamins, and metabolites of amino acids. Of 48 NHRs found in the human genome, several have already been known to play important roles not only in the development of primary and secondary lymphoid organs, but also in the differentiation and function of immune cells, including DCs, macrophages, and T cells (11–13). Sex hormones (e.g., estrogen and progesterone) and their receptors (e.g., estrogen receptor α: ERα, estrogen receptor β: ERβ, and progesterone receptor: PR) have long been known to have a profound influence on host immune response to microbial infections in the VM and lower female genital tract (14–16).

In addition to ER and PR which are the major female sex hormone receptors, several other NHRs are also associated with the modulation of proinflammatory gene repression and host immune responses. These include peroxisome proliferator-activated receptors (PPARs) (17, 18), vitamin D receptors (VDRs) (19, 20), retinoic acid receptor (RAR) (21), retinoid X receptor (RXR) (22), Nur77 (23), and liver X receptors (LXRs) (13, 22, 24–26). Nonetheless, except for limited data on the expression of ERα or PR by human VM APCs (27) and tissues (28–32), it is still unknown which other NHRs are expressed by different subsets of VM APCs, including DCs. Assessment of NHRs expressed by the APC subsets in the human VM will thus be the first step to understanding the potential roles of NHRs and their ligands in the immunology of human VM and lower female genital tract.

This study investigated which NHRs are expressed by individual subsets of the VM APCs and compared them with those expressed by APCs in human skin and blood. We further identified the major cell types expressing female sex hormone receptors, ERα and PR, in the human VM tissues. Data generated in this study will be fundamental for our understanding of the immunology of human VM in the future.

## 2 Materials and methods

### 2.1 Tissues and blood samples

Vaginal tissues were obtained from female adult patients who underwent pelvic or cosmetic surgeries. Abdominal skin tissues from female donors were also collected under protocols approved by the Institutional Review Board (IRB). Tissues were not procured from any individual who was pregnant or infected with HIV, hepatitis C virus, or tuberculosis. Tissues with any severe acute inflammation (with or without microbial infections) were also excluded. Severe acute inflammation at the time of surgery was determined by the surgeon’s evaluation of tissue redness, swelling, and pain.

Blood from healthy female donors was collected under the IRB protocol approved with written informed consent. Blood samples were not from the same patients who provided tissues, as vaginal tissues and abdominal skin tissues were not from the same patients. Blood samples were not procured from any individuals who were pregnant or infected with HIV, hepatitis C virus or tuberculosis. None of the tissue or blood donors was under any therapy with hormones for at least 6 months before the time of tissue collection. The demographic information of study participants is summarized in **Supplementary Table 1**. The numbers of tissues and blood samples used in each experiment and donor information are indicated in methods and figure legends. All experiments were performed in accordance with relevant guidelines and regulations.

### 2.2 Isolation of APC subsets

Both VM and skin tissue samples were processed as previously described (10, 27). In brief, epithelial sheets and LP pieces were separated after overnight treatment of tissues with Dispase type 2 (Roche Applied Science), and further incubated for 2 days before isolating single cells by Ficoll-sodium diatrizoate gradient (Lymphocyte Separation Medium, MP Biomedicals). Cells were then stained with antibodies and sorted with FACS Aria (BD Biosciences), as previously described (6, 10). Peripheral blood mononuclear cells (PBMCs) from age-matched healthy female volunteers were isolated by density gradient centrifugation using Ficoll-Paque PLUS (GE Healthcare). Blood myeloid DC (mDC, Lin1^-^HLA-DR^+^CD11c^+^CD123^-^) were enriched (PanDC kit, Stemcell) and sorted (6, 10).

To isolate VM APC subsets, single cell suspension of VM tissues was stained with 7-AAD (Biolegend), anti-CD14 (IgG1, 61D3, eBioscience), anti-CD1c (IgG1, L161, BioLegend), anti-Langerin (IgG2b, 15B10, in-house), anti-HLA-DR (IgG2a, L243, BioLegend). HLA-DR^+^ cells were gated and Langerin^+^ LCs, CD1c^+^CD14^-^ DCs, CD1c^+^CD14^+^ DCs, and CD1c^-^CD14^+^ MØ were sorted by FACS Aria II (BD Biosciences) (**Supplementary Figure 1**). HLA-DR^-^ cells were also sorted. To isolate skin DC subsets, a single cell suspension of skin biopsies was stained with the same antibodies used for the isolation of VM APC subsets. sLCs from the epidermis as well as CD1c^+^CD14^-^ DCs and CD1c^+^CD14^+^ DCs from the dermis were sorted by FACS Aria II (BD Biosciences). To isolate blood mDCs, PBMCs were stained with 7-AAD, Lin1 (BD Biosciences), anti-CD123 (IgG1, 9F5, BD), anti-CD11c (IgG1, B-ly6, BD) and anti-HLA-DR (IgG2a, L243, BioLegend). mDCs (Lin1^-^HLA-DR^+^CD11c^+^CD123^-^) were then sorted by FACS Aria (BD Biosciences).

For transcriptomics analysis, 8 different APC subsets were isolated from VM tissues, skin, and blood. Four APC subsets were isolated from the VM tissues of 41 patients. The numbers of individual APC subsets isolated were variable among tissues from different donors. Due to the small number of cells isolated from VM tissues, most samples tested in this study were prepared by combining the same cell types isolated from at least two tissue donors. A total of 48 samples were prepared with vaginal APC subsets. These include 9 samples of vLC isolated from 26 donors, 12 samples of vCD14^-^ DC isolated from 35 donors, 13 samples of vCD14^+^ DC isolated from 41 donors, and 14 vMØ isolated from 37 donors. It was of note that the majority of cDCs in lamina propria of human VM tissues were CD1c^+^ which could represent cDC1. Three skin DC subsets were isolated from tissues of 10 patients. We had 9 samples of sCD14^-^ DC, 8 samples of sCD14^+^ DC, and 10 sLC samples (total of 27 samples). Blood mDC from 6 female donors were isolated (6 samples). HLA-DR^-^ (after depletion of CD3^+^ T, CD19^+^/CD20^+^ B, and CD56^+^ NK cells) and HLA-DR^+^ non-APCs (**Supplementary Figure 1**) in VM tissues were also isolated from indicated numbers of tissue donors, as previously described (27).

### 2.3 Transcriptomics data analysis and statistical analysis

Methods for mRNA preparation, hybridization, data processing, and batch correction for the transcriptomics analysis were previously described (10). In brief, total RNA was isolated from cell lysates using the ArrayPure-Nano-scale RNA Purification Kit (Epicentre, Madison, WI). Agilent 2100 Bioanalyzer (Agilent, Palo Alto, CA) was used to measure RNA Integrity Numbers (RIN) for each sample. All samples with RIN values greater than seven were retained for further processing. RNA concentration was measured using a Nanodrop 1000 (Nanodrop Technologies, Wilmington, DE). 250 ng of RNA from all samples passing quality control were amplified and labeled using the TargetAmp™ 2-Round aRNA Amplification Kit 2.0 (Epicentre). 750 ng of amplified labeled RNA were hybridized overnight to Illumina HT12 V4 beadchips (Illumina, San Diego, CA). Chips were scanned on an Illumina BeadStation 500 following the manufacturer’s protocols. To identify technical sources of variability, we conducted principal variance component analysis (PVCA) using the PALO list of 27,935 expressed genes, which identified a significant source of batch effect between samples run in batches 1, 2, 3, and 4. Of note, batch 3 samples only contained vaginal DC or macrophages, and batch 4 only contained mDC and vaginal HLA-DR^-^ cells. To correct for this batch effect, we conducted ComBat correction using R/Bioconductor. One-way Welch analysis of variance (ANOVA) was conducted using an adjusted *P*-value cutoff of 0.05 with Benjamini-Hochberg multiple testing correction.

The weighted average proportion variance was calculated with the R/Bioconductor package ‘pvca’ (version 1.0.0) [http://www.bioconductor.org/packages/release/bioc/html/pvca.html]. The threshold used for the minimum amount of variance explained by the selected principal components was 0.5. The entire transcriptomics dataset described in this manuscript is deposited in the NCBI Gene Expression Omnibus [http://www.ncbi.nlm.nih.gov/geo] (GEO series accession number GSE54480). Statistical significance was determined using the analysis of variance test, as indicated in individual figure legends, using the Prism 8 software (GraphPad Software). Significance was set at *p*<0.05.

### 2.4 RT-PCR

Total RNAs from indicated cell types were isolated by using the RNeasy Mini Kit (Qiagen). Real-time polymerase chain reaction (PCR) was performed. SYBR Green qPCR Master Mix (Thermo Fisher) was used for amplifying *ESR1 and PGR*. Primer sequences were 5′-GAATCTGCCAAGGAGACTCGC-3′ and 5′-ACTGGTTGGTGGCTGGACAC-3′ for *ESR1* (protein ERα) and 5′-TGCTCAAGGAGGGCCTGCCGCAGGT-3′ and 5′-CTACTGAAAGAAGTTGCCTCTCGCC-3′ for *PGR* (protein PR). Target gene transcripts were normalized to the level of β2-microglobulin (B2M) mRNA. Amplification was carried out for 35 cycles of denaturation at 94°C, annealing at 55°C, and extension at 72°C.

### 2.5 Immunohistochemistry and microscopy

Cryosections were fixed in cold acetone, dried, and blocked for nonspecific fluorescence with an Fc receptor blocker and background buster (Innovex Biosciences). Sections were stained with the indicated Abs. Digital images were taken using an Olympus BX51 microscope utilizing the Planapo20/0.7 or Planapo40/0.95 objective, Roper Coolsnap HQ camera (Olympus), and Metamorph software (Molecular Devices). Confocal images were taken with the Leica SP1 (Leica) utilizing the Planapo63/1.32 objective. Images were acquired using the same exposures for antibody and isotype staining and identical scaling was applied.

Mouse anti-human ERα (IgG1, 6F11, Abcam) and rabbit anti-human PR (IgG, SP2, Abcam) along with goat anti-mouse IgG, goat anti-rabbit IgG as secondary antibodies were used for staining ERα and PR, respectively. Anti-human PR (SP2, Abcam) recognizes both isoforms of PR (PR-A and PR-B). Mouse anti-CD10 (IgG1, HI10a, BD Biosciences), and mouse anti-vimentin (IgG1, RV202, Abcam), were used. Appropriate isotype antibodies were used for all tissue/cell stainings performed in this study.

### 2.6 Statistical analysis

Statistical significance was determined using the analysis of variance test, Pearson correlation analysis, and *t*-test as indicated in individual figure legends, using the Prism 8 software (GraphPad Software). Significance was set at *p* < 0.05.

## 3 Results

### 3.1 Differential expression of NHRs by APC subsets in VM, skin, and blood

**Figure 1:**
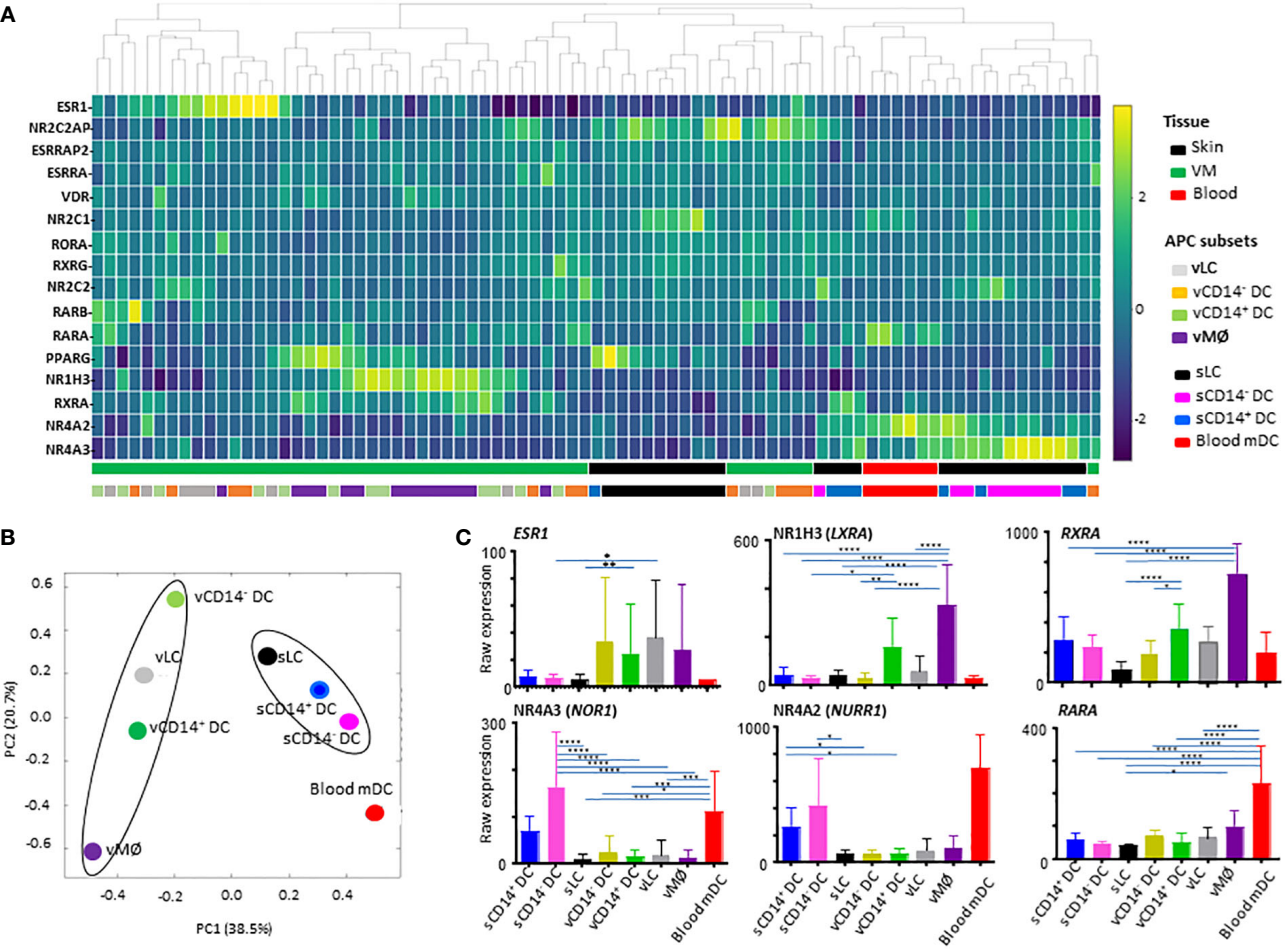
Analysis of nuclear hormone receptors (NHRs) expressed by antigen-presenting cell (APC) subsets isolated from human vaginal mucosa, skin, and blood. **(A)** Heatmap analysis of NHRs expressed by APC subsets (4 VM APC subsets, 3 skin APC subsets, and peripheral blood myeloid DCs). Unsupervised hierarchical clustering (Pearson correlation) of the 16 NHR transcripts differentially expressed between the 8 APC populations tested. **(B)** Principal variance component analysis (PVCA) for the nuclear hormone receptors differentially expressed by APC subsets. PVCA of the 81 samples analyzed, classified according to the normalized expression of transcripts identified in **(A)**. The samples are colored by 8 APC populations indicated. **(C)** Batch-corrected raw expression values for 6 NHR transcripts in **(A)** The expression of 10 additional NHR transcripts are presented in **Supplementary Figure 2**. Data are mean ± SD. Statistical analyses were performed with One-way ANOVA. n.s.: not significant, *p < 0.05, **p < 0.01, ***p < 0.005, ****p < 0.001.

The 81 samples obtained from VM tissues (48 samples), skin (27 samples), and blood (6 samples) were normalized to the median of all samples. With one-way ANOVA, we identified 16 differentially expressed transcripts (DETs) of NHRs among 81 samples for the 8 different APC subsets isolated from VM (4 subsets), skin (3 subsets), and peripheral blood (1 subset, mDC). Those 16 NHR transcripts were hierarchically clustered and presented as a heatmap in **Figure 1A**. We found that samples were primarily clustered by tissues of origin and then by different cell populations. Tissue-specific clustering was further substantiated by PVCA analysis (**Figure 1B**). vLC, vCD14^-^ DC, and vCD14^+^ DC clustered together in the VM, while sLC, sCD14^+^ DC and sCD14^-^ DC clustered in the skin. vMØ were also closer to vCD14^+^ DC than skin APCs and blood mDC. Blood mDC were closer to skin APC subsets than vaginal APC subsets.

To compare the expression levels of individual NHRs in different subsets of APCs tested, raw expression data are presented in **Figure 1C** and **Supplementary Figure 2**. Of note, *ESR1* (NR3A1) expression was detected mainly in the VM APC subsets. However, *ESR1* expression level was highly variable among individual donors, as it could be influenced by the hormonal status of patient donors (28, 33–35) which is also related to age, menstrual cycle, and menopausal status. *ESR2* or *PGR* expression was not detected. CD14^+^ vDC and vMØ, expressed increased levels of NR1H3 (*LXRA*, oxysterol receptor) and *RXRA* (NR2B1) (**Figure 1C**). vMØ also expressed increased levels of *ESRRA, VDR*, and *PPARG* when compared to most of the other APC subsets tested (**Supplementary Figure 2**).

We also found that skin APCs and blood mDC expressed increased levels of both NR4A3 (*NOR1*) and NR4A2 (*NURR1*)*. RARA* expression was also higher in blood mDCs than in other APC subsets tested (**Figure 1C**). *RARA/RXRA/PPAR* and *VDR* are known to contribute to vitamin A- and D-mediated immune modulation, respectively (20).

Taken these data together, we concluded that NHR expression profiles in the 8 different human APC subsets are mainly dependent on the tissues of origin. This highlights the potential roles of VM tissue-specific microenvironments in the expression of specific NHRs that might further influence the immune response in the VM and lower female genital tract.

### 3.2 Differential expression of NHRs by APC subsets and HLA-DR^-^ cells in the VM tissues

**Figure 2:**
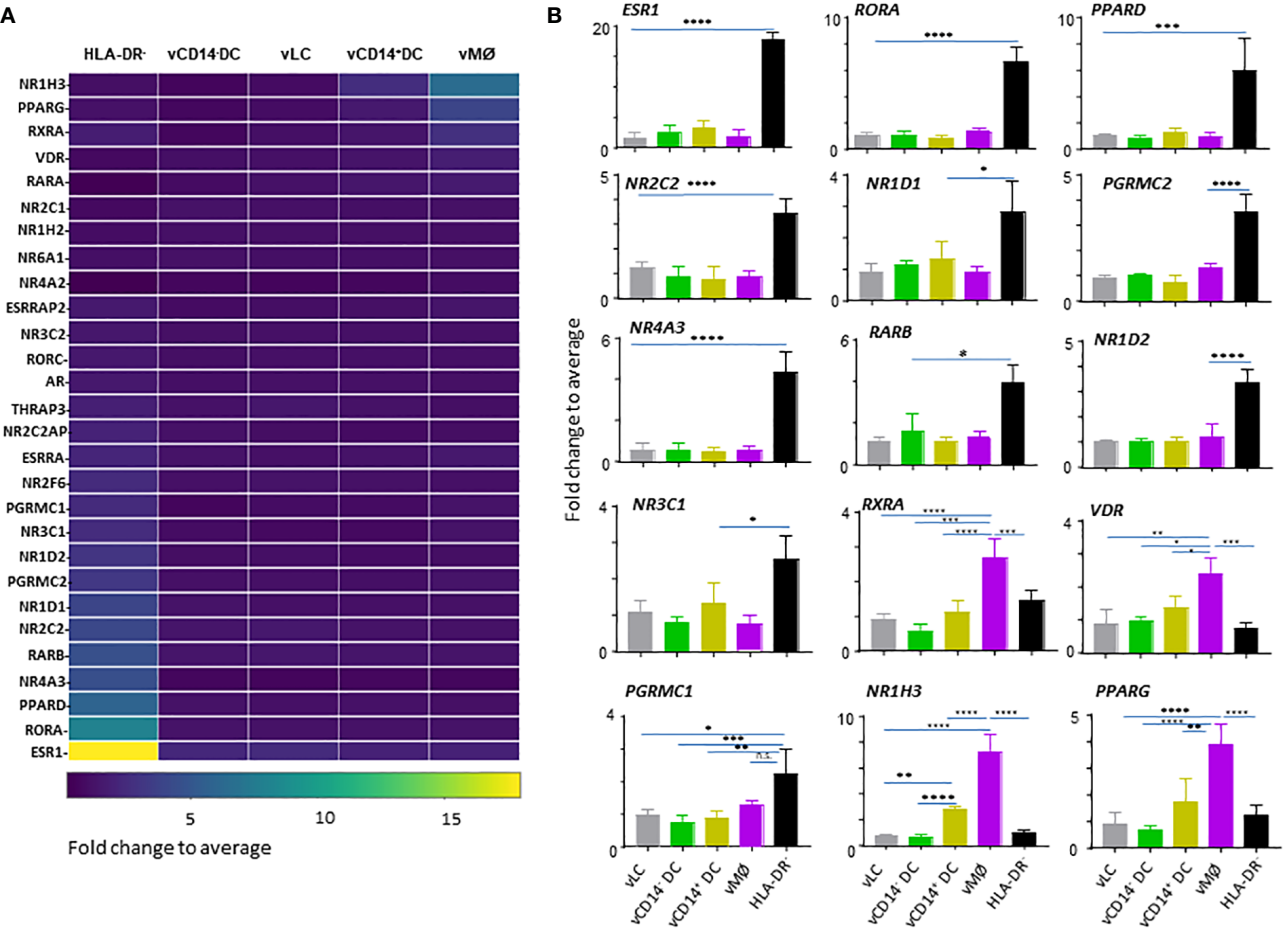
HLA-DR^-^ cells in the vaginal tissues express a large array of nuclear hormone receptors (NHRs). **(A)** Heatmap analysis of NHRs expressed by APC subsets (4 VM APC subsets and HLA-DR^-^ cells in the VM tissues). Unsupervised hierarchical clustering (Pearson correlation) of the 28 NHR transcripts differentially expressed between the 5 populations indicated. **(B)** Batch-corrected expression values for 15 represented NHR transcripts selected in **(A)**. Mean ± SD. Statistical analyses were performed with One-way ANOVA. n.s.: not significant, *p < 0.05, **p < 0.01, ***p < 0.005, ****p < 0.001.

We next assessed NHRs that are differentially expressed by the 4 VM APC subsets and HLA-DR^-^ cells isolated from VM tissues of 7 donors. A total of 49 samples obtained from vaginal tissues was normalized to the median of all samples. One-way ANOVA identified 28 DETs of NHRs among the five populations (HLA-DR^-^ cells and 4 VM APC subsets) (**Figure 2A**).

Although *ESR1* (NR3A1) expression was detected mainly in the VM APC subsets, when compared to APCs isolated from skin and blood (**Figure 1**), we found that HLA-DR^-^ cells, not the vaginal APCs, are the major cell types expressing *ESR1* (**Figure 2A, B**). In addition to *ESR1*, HLA-DR^-^ cells were also the major cell types expressing *RORA* (NR1F1), *PPARD* (NR1C2), *NOR1* (NR4A3), *RARB* (NR1B2), NR2C2 (*TR4*)*, NR1D1 (NR1D1), PGRMC2, NR1D2, NR3C1* (GCR), and *PGRMC1*. Of the VM APC subsets, vMØ expressed higher levels of NR1H3 (*LXRA*)*, PPARG* (NR1C3*), RXRA* (NR2B1), and *VDR* (NR1I1) than HLA-DR^-^ cells. Thyroid hormone receptors, NR1A1 (*THRA*) or NR1A2 (*THRB*), or androgen receptor (*AR*, NR3C4) expression was not observed. We could not detect two other major receptors for female sex hormones, *PGR* (NR3C3) and *ESR2* (NR3B2).

Taken these data together, we concluded that HLA-DR^-^ cells are the major cell types expressing a variety of NHRs in the human VM tissues. Except for the increased expression of *VDR* by vMØ, most of the steroid hormone receptors detected were also mainly expressed by HLA-DR^-^ cells, suggesting the potential roles of HLA-DR^-^ cells in the types and magnitude of immune response elicited in the VM and lower female genital tracts in response to such hormones. In addition, future studies will also need to investigate the potential roles of NR1H3*, PPARG, RXRA*, and *VDR* in the immune response elicited by vMØ.

### 3.3 Cell type- and tissue-specific expression of estrogen and progesterone receptors

**Figure 3:**
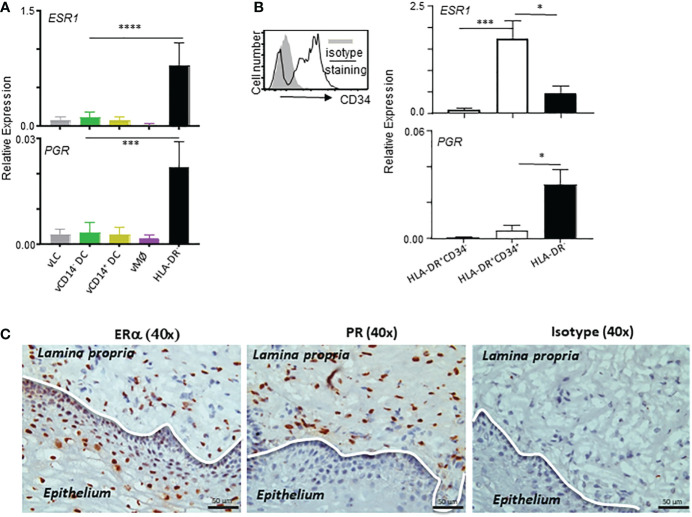
*ESR1* and *PGR* are expressed mainly by HLA-DR^+^CD34^+^ and HLA-DR^-^ cells, respectively, in the vaginal tissues. **(A, B)** qPCR analyses for *ESR1* and *PGR* expression (normalized to *B2M* expression levels) in indicated cell types. Samples tested in this experiment were collected from tissues from 9 donors (ages: 28, 32, 41, 58, 59, 64, 65, 72, and 75-year-old) were tested. Cells were stained and FACS-sorted as illustrated in **Supplementary Figure 1**. Data are presented with mean ± SD. Statistical analyses were performed with One-way ANOVA. n.s.: not significant, *p < 0.05, ***p < 0.005, ****p < 0.001. **(C)** Estrogen receptor alpha (ERα) and progesterone receptor (PR) expression in the human vaginal mucosal (VM) tissues. Immunohistochemical staining (x40) of frozen VM tissues with anti-ERα, anti-PR, and isotype control antibodies. Data are representative of experiments performed with tissues from 10 different patients.

Female sex hormones are known to play important roles in the immunity in the VM and lower female genital tract. We therefore further examined the expression levels of *ESR1* and *PGR* in the VM APC subsets and HLA-DR^-^ cells by qPCR (**Figure 3A**). In line with the data in **Figure 2**, *ESR1* expression was higher in HLA-DR^-^ cells than in any of the VM APC subsets. *PGR* expression was detectable by qPCR with expression by HLA-DR^-^ cells, though its expression level was lower than that of *ESR1*. Similar to *ESR1*, *PGR* expression was also higher in HLA-DR^-^ cells than in the VM APC subsets tested. Of note, approximately 90% of the total human VM tissues are HLA-DR^-^ cells (10) that contain stromal cells and smooth muscle cells in the LP of the VM tissues.

It is also important to note that the majority (more than 85-95%) of HLA-DR^+^ cells in the human VM tissues are not belonging to the VM APCs (expressing Langerin, CD1c, or CD14) (10). In addition, approximately 70% of HLA-DR^+^ non-APC populations expressed CD34 (**Supplementary Figure 1**). We found that *ESR1* expression was higher in HLA-DR^+^CD34^+^ cells than HLA-DR^-^ cells, whereas *PGR* expression was higher in HLA-DR^-^ cells than HLA-DR^+^CD34^+^ cells. No significant expression of *ESR1* or *PGR* was detected in HLA-DR^+^CD34^-^ cells. These data indicate that the differential outcomes of the actions of estrogen and progesterone (14–16) could be, at least in part, due to their ability to target different cell types in the same tissue microenvironment. Both endothelial and epithelial cells in intestinal mucosal tissues were also previously reported to constitutively express HLA-DR (36–38). In support of the qPCR data in **Figure 3B**, ERα expression was detected in both epithelium and LP of the VM tissues, whereas PR expression was detected only in LP of the VM tissues (**Figure 3C**; **Supplementary Figure 3**).

**Figure 4:**
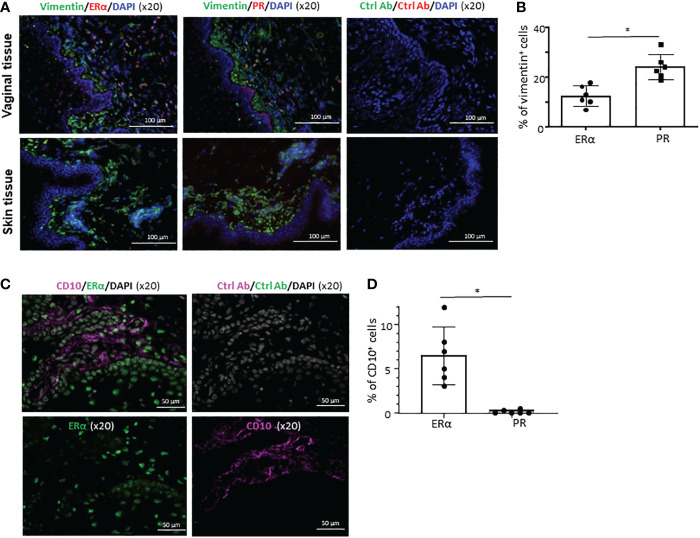
Cell type- and tissue-specific expression of ERα and PR. **(A)** Frozen sections of the VM tissues (upper panels) and abdominal skin tissues (lower panels) were stained with anti-vimentin antibody along with anti-ERα or anti-PR antibody (x10). Tissue sections were also stained with isotype control antibodies. Ages of VM and skin tissue donors were 33 and 38 year-old, respectively. Data generated with VM tissue from 3 other donors (49, 59, and 62-year-old) and skin tissues from 2 other donors (48 and 58-year-old) showed similar results. **(B)** Data (mean ± SD) from two independent experiments using tissues from 6 donors (33, 43, 60, 68, 71, and 72-year-old) are presented. **(C)** Frozen sections of the VM tissues (from 43-year-old) were stained with anti-CD10 along with anti-ERα or anti-PR antibody (x20). Tissue sections were also stained with isotype control antibodies. VM tissues of two other donors (37 and 69-year-old) showed similar results. **(D)** Data (mean ± SD) from three independent experiments using tissues from 6 donors (29, 49, 51, 59, 63, 68-year-old) are presented. Statistical significance was determined with a paired t-test. *p < 0.05.

Similar to our previous observation (27), a significant fraction of cells in the VM tissues expressing ERα or PR co-expressed vimentin (upper panels in **Figures 4A, B**) that is known to be expressed in a variety of cell types, including smooth muscle cells and stromal cells (39, 40). However, neither ERα nor PR was detected in human skin (lower panels in **Figure 4A**). This is in line with the data published in a previous study (41), showing that protein ER or PR expression was not detected in normal human skin. This also supports an idea for a tissue-specific action of female sex hormones *via* ERα and PR in a steady state. We further found that some of the ERα^+^ cells in the LP of VM tissues express CD10 (**Figure 4C**). None of the ERα^+^ cells in the epithelium expressed CD10. No significant colocalization of CD10 and PR were observed (not shown). Summarized data of the percentage of CD10^+^ERα^+^ in total ERα^+^ cells are presented in **Figure 4D**. Taken all these data together, we concluded that ERα and PR expression is specific for not only cell types, but also tissues of origin.

## 4 Discussion

This study investigated NHR expression profiles in 4 different human VM APC subsets by comparing them with those of 4 other APC subsets isolated from human skin and peripheral blood. A total of 16 differentially expressed NHR transcripts were found, and their expression profiles were primarily related to tissues of origin, and then APC subsets to a less degree. Importantly, this study also reports that HLA-DR^-^ cells (approximately more than 90% of total VM tissue cells) are the major cell types expressing a large array of NHRs, including several steroid hormone receptors. Further analyses of the cell types isolated from the human VM tissues revealed that *ESR1* (ERα) and *PGR* (PR) expressions are enriched mainly in HLA-DR^+^CD34^+^ and HLA-DR^-^ cells, respectively. These new data support the idea that differential effects of female sex hormones (14–16, 42) (e.g., estrogen versus progesterone) could be due to, at least in part, by their actions on different cell types in the different tissue microenvironments. Furthermore, NHR expression data reported in this study were generated with cells directly isolated from human tissues and could thus be a fundamental basis for future studies of the roles of NHRs and their ligands in shaping host immune response not only in local tissues, especially for VM and lower female genital tract, but also systemically.

Critical roles of certain NHRs (PPARs, VDR, RAR, RXR, Nur77, LXRs) (13, 17–26) and their ligands in host immune response were previously reported. Previous studies (11, 43–46) also reported potential roles of NHRs expressed by human DCs in host immune response. However, it is important to note that many of the previous studies were performed with monocyte-derived *in vitro* generated DCs (11, 44–46) that may not be the same as *in vivo* DCs. Not surprisingly, ERα and PR expression by certain cell types in the human VM tissues is not constitutive, but inducible (27). In this regard, data from this study using DCs directly isolated from the human VM tissues, in comparison with DCs from skin and blood, will be fundamental for our understanding of the expression of NHRs and their potential roles in immune response elicited in the human VM tissues.

Of the 16 NHRs found in **Figure 1**, the *ESR1* expression level in the VM APC subsets was highly variable among donors. Such variation in *ESR1* expression could be related to the hormonal status of patients that is influenced by age, menstrual cycle, and menopausal state of patients (27, 28, 33–35, 47). These factors might also relate to the donor variations for the expression levels of other NHRs. In contrast to the APC subsets, however, the expression levels of NHRs in HLA-DR^-^ cells were less variable among donors. These data are in line with our previous data (27), showing that ERα was constitutively expressed in non-APC cell types in the human VM tissues, whereas ERα expression in the VM APCs and some other cell types was inducible. It also indicates the potential roles of such HLA-DR^-^ cell types in the mechanisms of action of such hormones that need to be further studied in the future. It is also important to note that estrogen and progesterone may act on target cells *via* other receptors (14, 42, 48–51), even though ER and PR are their major receptors. Indeed, progesterone can act on T cells *via* membrane progesterone receptors (52) as well as glucocorticoid receptors (53).

qPCR data generated with the 4 VM APC subsets further demonstrated that any of the VM APCs are not the major cell types expressing *ESR1* or *PGR*. This study also found that the major cell types expressing *ESR1* and *PGR* are not the same in the human VM tissues, *ESR1* expression by CD34^+^HLA-DR^+^ cells and *PGR* expression by HLA-DR^-^ cells. This indicates that the differential effects of estrogen and progesterone on the magnitude and types of immune response could be at least in part their ability to target different cell types in the VM tissue microenvironment. Future study needs to investigate how CD34^+^HLA-DR^+^ and HLA-DR^-^ cells contribute to the immune response in the VM in response to estrogen and progesterone, respectively. Interestingly, a large fraction of the ERα^+^ cells in the LP of the VM tissues co-expressed vimentin and CD10 which are also known to be expressed by stroma and smooth muscle cells (39, 40). In addition, those non-immune ERα^+^ and/or PR^+^ cells in the human VM tissue sections did not co-express CD31, CD44, CD56, CD66, CD117, CD324, or E/P selectins that could be expressed by endothelial cells and epithelial progenitor cells (not shown).

In summary, this study reports for the first time that VM APC subsets express distinct profiles of NHRs when compared to APC subsets in skin and blood. In addition, the majority of NHRs detected in the human VM tissues were mainly expressed by HLA-DR^-^ cells which are the major cell type (approximately 90% of the total) in the human VM tissues. Importantly, ERα and PR expression in the VM tissues are highly specific to cell types, e.g., ERα expression by HLA-DR^+^CD34^+^ cells and PR expression by HLA-DR^-^ cells. Although future studies with well-defined tissue donors (e.g., age, menstrual cycle, menopausal status) (28, 33–35) could provide additional information, data from this study are fundamental bases for our understanding of the immunology of human vagina in the future.

## Data availability statement

The datasets presented in this study can be found in online repositories. The names of the repository/repositories and accession number(s) can be found in the article/**Supplementary Material**.

## Ethics statement

The studies involving human participants were reviewed and approved by Institutional Review Board, Mayo Clinic, Baylor Research Institute. The patients/participants provided their written informed consent to participate in this study.

## Author contributions

HJ, CG, MW, EF, JY, and SO designed experiments, analyzed data, and wrote the manuscript. HJ, CG, DD, MW, EF and VN performed experiments. All authors contributed to the article and approved the submitted version.
