# Supplementary material for: Differential expression of nuclear hormone receptors by dendritic cell subsets in human vaginal mucosa and skin
Source: Front Immunol. 2023 Jan 13;13:1063343. doi: 10.3389/fimmu.2022.1063343 (PMC9880315; doi:10.3389/fimmu.2022.1063343)
Supplement: Supplementary file 1 [file DataSheet_1.pdf]

Supplementary Material

Differential Expression of Nuclear Hormone Receptors by Dendritic Cell Subsets in Human Vaginal Mucosa and Skin

HyeMee Joo[1], Chao Gu[1], Matthew Wiest[1], Dorothee Duluc[2], Emyly Fernandez[1], Verah Nyarige[3], Johnny Yi[4], SangKon Oh[1]

[1]Department of Immunology, Mayo Clinic, 13400 E. Shea Blvd, Scottsdale, AZ
[2]Immunoconcept, CNRS UMR 5164, Bordeaux University, Bordeaux, France
[3]Department of Health Sciences Research, Mayo Clinic, 13400 E. Shea Blvd, Scottsdale AZ
[4]Department of Medical and Surgery Gynecology, Mayo Clinic, 5777 E. Mayo Blvd, Phoenix, AZ

This file includes:

Supplementary Figure 1. Gating strategy for the isolation of vaginal APC subsets (vLC, $vCD14^-DC$, $vCD14^+$ DC, and vMØ), $HLA\text{-}DR^-$ cells, and $HLA\text{-}DR^+CD34^+$ cells from the single cell suspension of vaginal tissues.

Supplementary Figure 2. Batch-corrected raw expression values for the 10 NHR transcripts in Figure 1A.

Supplementary Figure 3. Immunofluorescent staining (x10) of VM tissues with anti-ERα and anti-PR antibodies.

Supplementary Table 1. Information of study participants.

Supplementary Figure 1

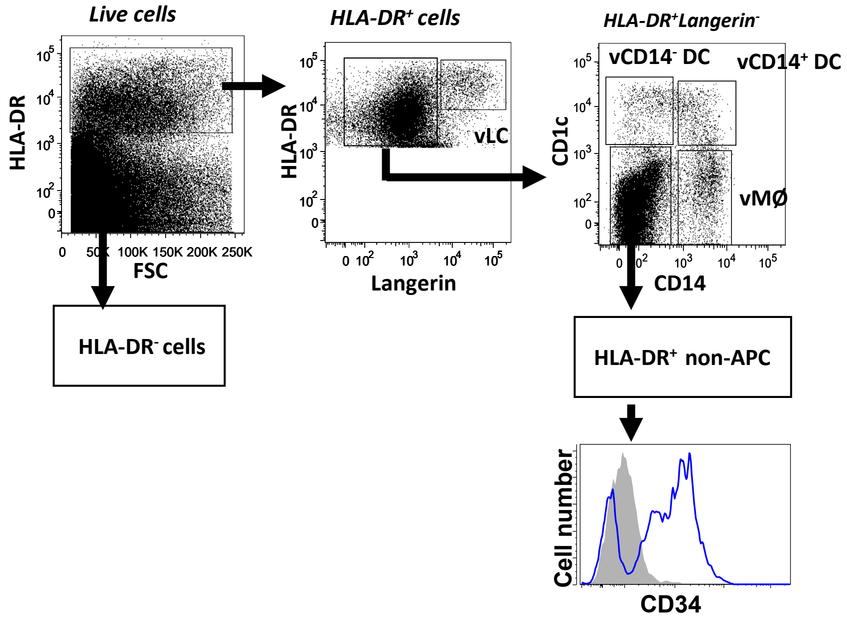

Supplementary Figure 1. Gating strategy for the isolation of APC subsets (vLC, vCD14-DC, vCD14+ DC, and vMØ), HLA-DR- cells, and HLA-DR$^+$CD34$^+$ cells from the single cell suspension of vaginal tissues.

Supplementary Figure 2

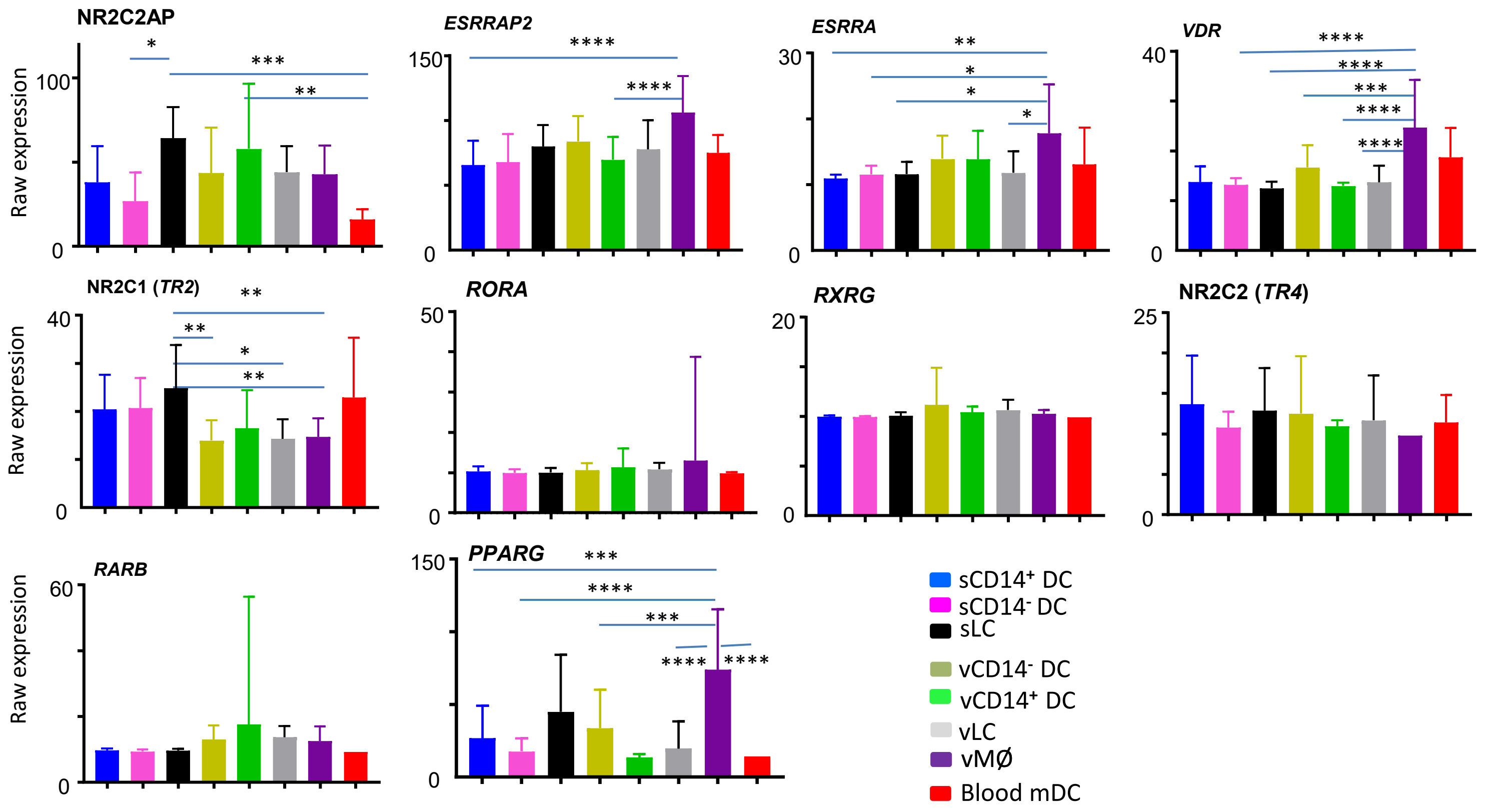

Supplementary Figure 2. Bar charts representing the batch-corrected raw expression values of the 10 NHR transcripts in **Figure 1A**. Mean ± SD. Statistical analyses were performed with One-way ANOVA. n.s. not significant, * p<0.05, ** p<0.01, *** p<0.005, **** p<0.001.

Supplementary Figure 3

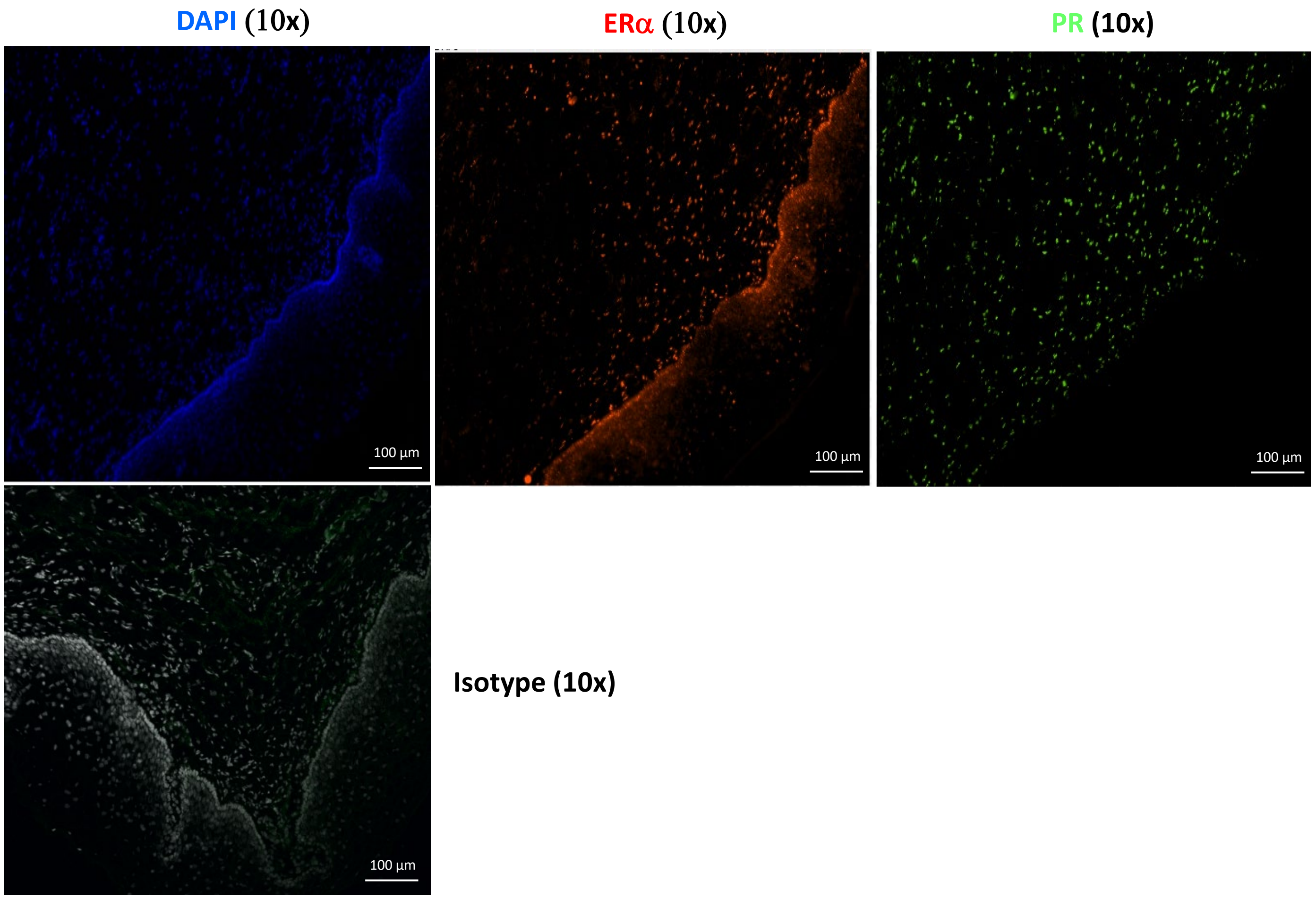

Supplementary Figure 3: Immunofluorescent staining (x10) of VM tissues with anti-ERα and anti-PR antibodies. Tissue sections were also stained with DAPI and isotype control antibodies. Data are representative of more than 10 independent experiments using tissues from 10 different patients.

Supplementary Table 1. Information of study participants

Participant Demography	Age (years)[a]
Vaginal tissue donors (N=68)	63 (28-75)
Hispanic (N=9)	59 (34-75)
Non-Hispanic (N=59)	65 (38-74)
White (N=49)	66 (28-75)
Black/African American (N=9)	57 (37-68)
Asian (N=3)	68 (53-71)
Others (N=7)	54 (39-67)
Skin tissue donors (N=13)	58 (37-66)
Hispanic (N=3)	61 (49-62)
Non-Hispanic (N=10)	57 (37-66)
White (N=9)	59 (37-66)
Black/African American (N=2)	52 and 59
Others (N=2)	43 and 62
Blood donors (N=6)	51 (25-64)
Hispanic (N=1)	37
Non-Hispanic (N=5)	49 (25-64)
White (N=4)	50 (25-60)
Others (N=2)	41 and 63

[a]Age is given as median (range).
